# Hydrophobization of Chitin Nanofibers by Grafting of Partially 2-Deoxygenated Amyloses Through Enzymatic Approach

**DOI:** 10.3390/molecules30010016

**Published:** 2024-12-24

**Authors:** Naoki Yamamoto, Masayasu Totani, Jun-ichi Kadokawa

**Affiliations:** Graduate School of Science and Engineering, Kagoshima University, 1-21-40 Korimoto, Kagoshima 890-0065, Japan; k9828683@kadai.jp (N.Y.); m-totani@cb.kagoshima-u.ac.jp (M.T.)

**Keywords:** chitin nanofiber, enzymatic grafting, glucan phosphorylase, hydrophobization, reductive alkylation

## Abstract

In recent years, increased attention has been given to the effective use of chitin nanofibers (ChNFs). We have developed a method to fabricate thinner chitin nanomaterials, called scale-down chitin nanofibers (SD-ChNFs), by a bottom-up procedure at the nanoscale level, with subsequent disintegration by electrostatic repulsion. The surface modification of SD-ChNFs is anticipated to provide new properties and functions for their practical applications. Inspired by our previous reports, which found hydrophobicity in partially 2-deoxygenated (P2D-) amylose obtained by the glucan phosphorylase (GP)-catalyzed enzymatic copolymerization of α-d-glucose 1-phosphate/d-glucal as comonomers, this work investigated the hydrophobization of SD-ChNFs via an enzymatic approach. After the modification of maltooligosaccharide primers on SD-ChNFs was performed by a reductive alkylation toward ChNFs, the grafting of the P2D-amyloses was performed by GP-catalyzed enzymatic copolymerization. ^1^H NMR analysis supported the production of P2D-amylose-grafted SD-ChNFs with different d-glucose/2-deoxy-d-glucose unit ratios on SD-ChNFs. The X-ray diffraction analysis of the products confirmed that the chain lengths and unit ratios of the grafted polysaccharides strongly affected the entire crystalline structures. Water contact angle measurements of the cast films of the products indicated that successful hydrophobization was achieved by the grafting of P2D-amylose chains with a sufficient chain length, a relatively high 2-deoxy-d-glucose unit ratio, and low crystallinity.

## 1. Introduction

Although chitin is the most abundant natural aminopolysaccharide, it remains underutilized due to its poor solubility, processability, and feasibility. This problem is caused by the characteristics of its chemical structure, mostly attributed to acetamido groups at the C-2 position repeating *N*-acetyl-d-glucosamine (GlcNAc) residues, leading to the formation of numerous intermolecular hydrogen bonds [1,2]. In order to overcome this issue, researchers have developed various chitin derivatives via modifications including acylation [3], cross-linking [4], and graft polymerization [5], which have the potential to exhibit unique functions, such as multidimensional properties, highly sophisticated functions, and broad application capacities in biomedical and other industrial fields [6,7].

Recently, chitin nanowhiskers and chitin nanofibers (ChNFs), obtained through top-down or bottom-up methods, have gained attention as functional materials [8,9,10] because they are valued in applications such as in food additives [11,12] and reinforcing agents [13,14] in polymer composites due to their safety, biodegradability, light weight, and strength [8,14,15,16]. The surface of ChNFs has been modified with desired substituents to improve properties and provide new functions, such as dispersibility in oil and rubber materials and suitableness in viscosity modifiers and reinforcing agents [17,18,19,20]. However, as there are still not many approaches to the surface modification of ChNFs, attempts to develop modification methods have been carried out, to fabricate new functional ChNF materials.

We have found that self-assembled ChNFs with a width of 20–60 nanometers and a length of several hundred nanometers are efficiently formed via the regeneration of chitin from its ion gel with 1-allyl-3-methylimidazolium bromide (AMIMBr, ionic liquid), using methanol [21]. Furthermore, the treatment of the self-assembled ChNFs with aqueous NaOH for partial deacetylation and subsequent dispersion in aqueous acetic acid by electrostatic repulsion among protonated amino (ammonium) groups gives rise to thinner nanofibers, called scale-down ChNFs (SD-ChNFs) [22]. Compared to the bio-based nanofibers, such as cellulose nanofibers, there are not many examples of modification and derivatization in ChNFs. We have previously reported different types of modification and grafting approaches to functionalizing the abovementioned self-assembled ChNFs and SD-ChNFs [19,23,24,25,26]. For example, we developed an efficient approach to the modification of naturally occurring monosaccharide and oligosaccharide substituents on the surfaces of self-assembled ChNFs and SD-ChNFs to fabricate chitin nanomaterials solely composed of saccharide chains, which was achieved through reductive alkylation using NaBH_2_CN as a reducing agent [25,27]. Additionally, after primers were modified by this method, glucan phosphorylase (GP)-catalyzed enzymatic polymerization, as described below, could be performed to graft amylose, a natural polysaccharide, onto the self-assembled ChNFs [28].

The grafting of polysaccharide chains onto polymeric backbones through an enzymatic approach represents an effective approach to imparting environmentally friendly and biological properties to the final products [29,30]. Moreover, the enzymatic approach has been identified as a useful tool with which to precisely synthesize well-defined polysaccharides, because the reaction occurs in highly controlled regio- and stereoselective arrangements. For example, the enzymatic polymerization of α-d-glucose 1-phosphate (Glc-1-P) as a monomer, catalyzed by glucan phosphorylase (GP), has been effectively applied to graft amylose chains onto various polymeric backbones. It operates with a high specificity to induce the propagation from the non-reducing end of a maltooligosaccharide primer, representatively given by the elemental reaction [α(1→4)-Glc]*_n_* + Glc-1-P → [α(1→4)-Glc]*_n_*_+1_ + inorganic phosphate, giving rise to a well-defined α(1→4)-glucan [31]. Thus, GP-catalyzed enzymatic polymerization using primers modified on appropriate polymeric backbones at their reducing ends (with no participation in the polymerization) is employed to precisely fabricate different types of amylose-grafted functional polymeric materials, e.g., chitin/chitosan, (carboxymethyl)celluloses, and alginate [5,32,33]. Notably, GP, isolated from thermophilic bacteria, exhibits weak specificity in substrate recognition, allowing the use of certain analog monomers of Glc-1-P (i.e., 1-phosphates of different monosaccharides from Glc) in the polymerization process to create unnatural polysaccharides (amylose analogs) [34,35,36,37,38,39]. We recently discussed the strong hydrophobicity of a film, produced by casting, prepared from an enzymatically synthesized partially 2-deoxygenated (P2D-) amylose with an amorphous property, created by a random fashion in the Glc/2-deoxyglucose (2dGlc) unit sequence. The synthesis of such an unnatural polysaccharide was achieved through the thermostable GP (from *Aquifex aeolicus* VF5)-catalyzed enzymatic copolymerization of Glc-1-P/d-glucal, initiated from maltotriose as a primer [40,41]. In this process, 2-deoxy-α-d-glucose 1-phosphate (2dGlc-1-P), which is generated in situ from d-glucal (involving a GP-catalyzed 1,2-addition of a non-reducing hydroxy group at the C-4 position of the primer to d-glucal and subsequent phosphorolysis), serves as the actual comonomer [40]. The hydrophobic behavior of this polysaccharide is likely attributed to the increasing number of hydrophobic regions owing to the absence of hydroxy groups at the C-2 positions of amylose [41,42]. Moreover, the enzymatic grafting of P2D-amylose chains onto hydrophilic bio-based polymers, such as carboxymethyl cellulose, glycogen, and poly(γ-glutamic acid), through thermostable GP catalysis has advantageously imparted hydrophobicity into the obtained P2D-amylose-grafted derivatives, with water contact angle values exceeding 100° [33,41,43,44]. Particularly, we reported that this enzymatic grafting method could be applied to fabricating P2D-amylose-grafted hydrophobic cellulose nanofibers [33]. Moreover, the hydrophobization approach of hydrophilic bio-based polymers can probably be performed while preserving their environmental friendliness and biodegradability, because 2-deoxyamylose, obtained by the GP-catalyzed enzymatic polymerization of d-glucal, has been reported to enzymatically be hydrolyzed by amylase [45].

**Figure 1:**
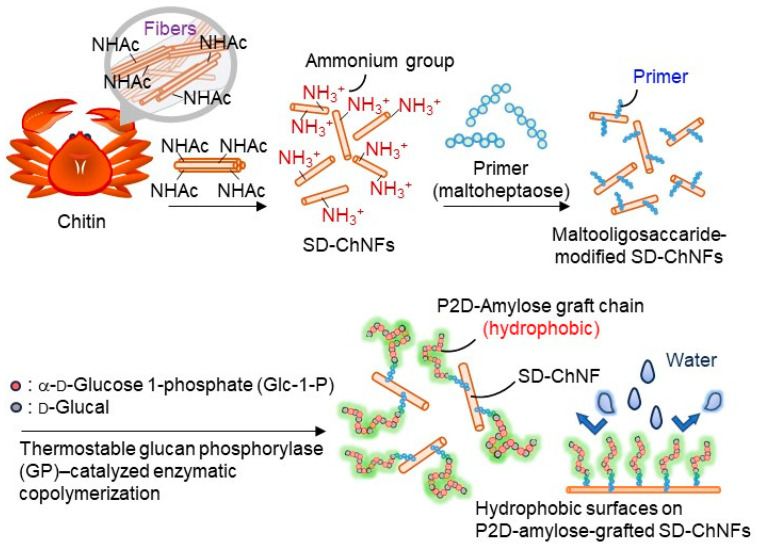
Schematic illustration of enzymatic grafting of partially 2-deoxygenated (P2D-) amylose on scale-down chitin nanofibers (SD-ChNFs) by thermostable glucan phosphorylase (GP) catalysis, as the concept of this study.

Based on the abovementioned background and viewpoint, the present study demonstrates the hydrophobization of SD-ChNFs by the grafting of P2D-amylose chains through the thermostable GP-catalyzed enzymatic copolymerization. We formulated two hypotheses: (1) enzymatic polymerization would be able to control polymer growth directly from the surface of SD-ChNFs; and (2) the composition ratio of P2D-amylose chains might significantly influence the crystalline and surface properties of the products. This includes the chemical modification of maltooligosaccharide primers on SD-ChNFs by reductive alkylation and the subsequent thermostable GP-catalyzed enzymatic copolymerization of Glc-1-P/d-glucal (Figure 1). Consequently, the present study will reveal that the enzymatic grafting approach of P2D-amylose chains effectively leads to the production of hydrophobic SD-ChNF films.

## 2. Results and Discussion

**Scheme 1:**
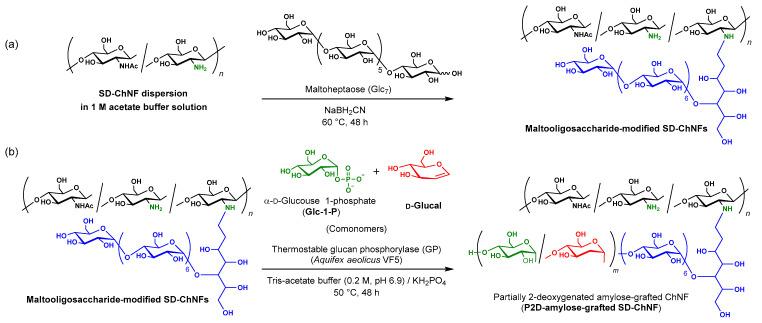
Reactions for (**a**) preparation of maltooligosaccharide-modified SD-ChNFs and (**b**) subsequent thermostable GP-catalyzed copolymerization of comonomers (Glc-1-P/d-glucal) to obtain P2D-amylose-grafted SD-ChNFs.

**Figure 2:**
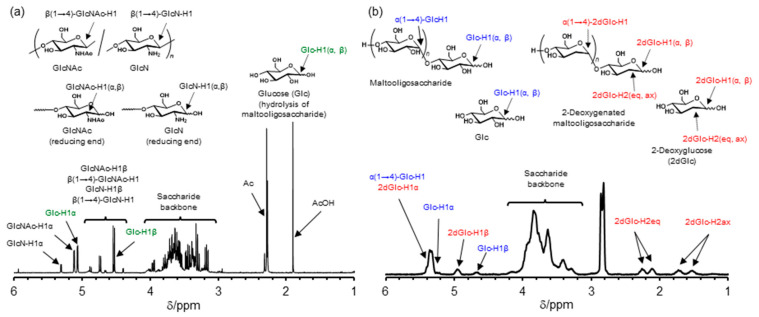
^1^H NMR spectra of (**a**) the sample after hydrolysis and dissolution of maltooligosaccharide-modified SD-ChNFs in DCl/D_2_O (=50/50 vol/vol), and (**b**) the sample after selective hydrolysis and dissolution of P2D-amylose graft chains on SD-ChNFs (entry 2, Table 1) in DCl/D_2_O/DMSO-*d*_6_ (=43/43/14 vol/vol/vol).

For the preparation of the SD-ChNFs, the PDA-ChNF film was first produced according to our previously reported procedure [25,46]. The degree of deacetylation (DDA) value of the resulting film was estimated from the integrated ratio of the anomeric signals at 4.5–5.2 ppm to the signal at 2.2–2.5 ppm derived from the acetamido group (-NHAc) and the signal at around 1.9 ppm derived from acetic acid (AcOH, produced by the partial hydrolysis of the acetamido groups) in the ^1^H NMR spectrum (after acidic hydrolysis in D_2_O/DCl; Figure S1) to be 31%. The modification of the maltooligosaccharide primer on the SD-ChNFs was then carried out through reductive alkylation with maltoheptaose based on our procedure, as previously reported (Scheme 1a) [28]. In the ^1^H NMR spectrum of the sample after acidic hydrolysis and the dissolution of maltooligosaccharide-modified SD-ChNFs in D_2_O/DCl (=50/50 vol/vol), two reducing anomeric signals (Glc-H1β and Glc-H1α) derived from the maltooligosaccharides were observed at 4.38 and 5.07 ppm, besides anomeric signals assignable to GlcNAc and d-glucosamine (GlcN) residues, derived from the chitin main-chain (Figure 2a), supporting the successful modification of the maltooligosaccharide primers on SD-ChNFs.

The degree of substitution (DS) of maltooligosaccharide primers on SD-ChNFs was estimated from the integrated ratio of the reducing anomeric signals to the main-chain anomeric signals to be 24%. The thermostable GP-catalyzed enzymatic copolymerization of Glc-1-P/d-glucal (comonomers) from the primers modified on SD-ChNFs was then performed in Tris-acetate buffer (0.20 M, pH 6.9) containing KH_2_PO_4_ for 48 h at 60 °C to synthesize the P2D-amylose-grafted SD-ChNFs. The different Glc-1-P/d-glucal feed ratios were taken to vary Glc/2dGlc unit ratios in the grafted P2D-amylose chains (entries 2–6 in Table 1, Scheme 1b).

The products of entries 3–6 were precipitated after the copolymerization reaction at 60 °C for 48 h, which were thus isolated by suction filtration and dried for 24 h at 60 °C under reduced pressure. In contrast to these products, the product from entry 2 was not precipitated after the reaction. Therefore, the reaction solution was additionally cooled at 4 °C for 24 h and then centrifuged to obtain a flocculent material. The different behavior of the product of entry 2 from others was probably attributed to its low crystallinity, as discussed in the following powder X-ray diffraction (XRD) results. The amylose- and 2-deoxyamylose-grafted SD-ChNFs (entries 1 and 7 in Table 1) as the reference grafted materials were also synthesized through the thermostable GP-catalyzed enzymatic homopolymerizations of Glc-1-P and d-glucal as monomers by the same operations, respectively. Figure 2b shows the ^1^H NMR spectrum of the sample after the selective hydrolysis and dissolution of the P2D-amylose chains (entry 2) in D_2_O/DCl/DMSO-d_6_, grafted on SD-ChNFs, because the product was insoluble in common NMR solvents. The structure of the product was supported by the signals’ assignments as shown in Figure 2b and described in the Materials and Methods section. Particularly, the ^1^H NMR spectrum newly observed the characteristic signals derived from the 2dGlc residues, i.e., 2dGlc-H2 signals in axial and equatorial positions at 1.53/1.65 and 2.10/2.25 ppm, respectively, and anomeric signals ascribed to 2dGlc-H1β and 2dGlc-H1α (reducing ends) at 4.95 and 5.23–5.42 ppm, respectively, in addition to the anomeric signals derived from Glc residues, similar to those detected in Figure 2a. The ^1^H NMR spectra of the samples, acidically hydrolyzed from the products of entries 4–7, are shown in Figures S3–S8, which have exhibited similar patterns to those in Figure 2b. These ^1^H NMR results sufficiently suggested the construction of the P2D-amylose-grafted SD-ChNFs through the enzymatic copolymerization in different comonomer feed ratios listed in Table 1. The Glc/2dGlc unit ratios in the P2D-amylose graft chains, which were evaluated from the integrated ratios of the anomeric signals assigned to the two units, were according to the Glc-1-P/d-glucal feed ratios (entries 2–6 in Table 1). The ^1^H NMR spectra of the products by the homopolymerizations of Glc-1-P and d-glucal (entries 1 and 7 in Table 1) also supported the production of the amylose and 2-deoxyamylose-grafted SD-ChNFs, as shown in Figures S3 and S8, respectively.

The degrees of polymerization (DPs) of the saccharide graft chains on SD-ChNFs were estimated based on the weight yields of the products, the Glc/2dGlc unit ratios, and the DS of the maltooligosaccharide primer on SD-ChNFs. The DP values of the amylose and 2-deoxyamylose graft chains were 47 and 3, respectively (entries 1 and 7, Table 1). The DP values of P2D amylose graft chains of entries 3–6 in Table 1 tended to decrease, i.e., 30, 28, 27, and 22, as the ratios of 2dGlc units increased. This is probably because of the weaker recognition ability of d-glucal than Glc-1-P by thermostable GP, resulting in difficulty in enzymatic chain-elongation. We also found similar tendencies depending on the Glc-1-P/d-glucal feed ratios in the case of the thermostable GP-catalyzed grafting of the P2D-amylose chains on carboxymethyl cellulose, glycogen, poly(γ-glutamic acid), and cellulose nanofiber [33,41,43,44]. The specific lower DP value in the product of entry 2 than that of entries 3–5, in spite of the lower 2dGlc feed ratio, was probably due to the difficulty in precipitation from the reaction mixture as mentioned above.

**Figure 3:**
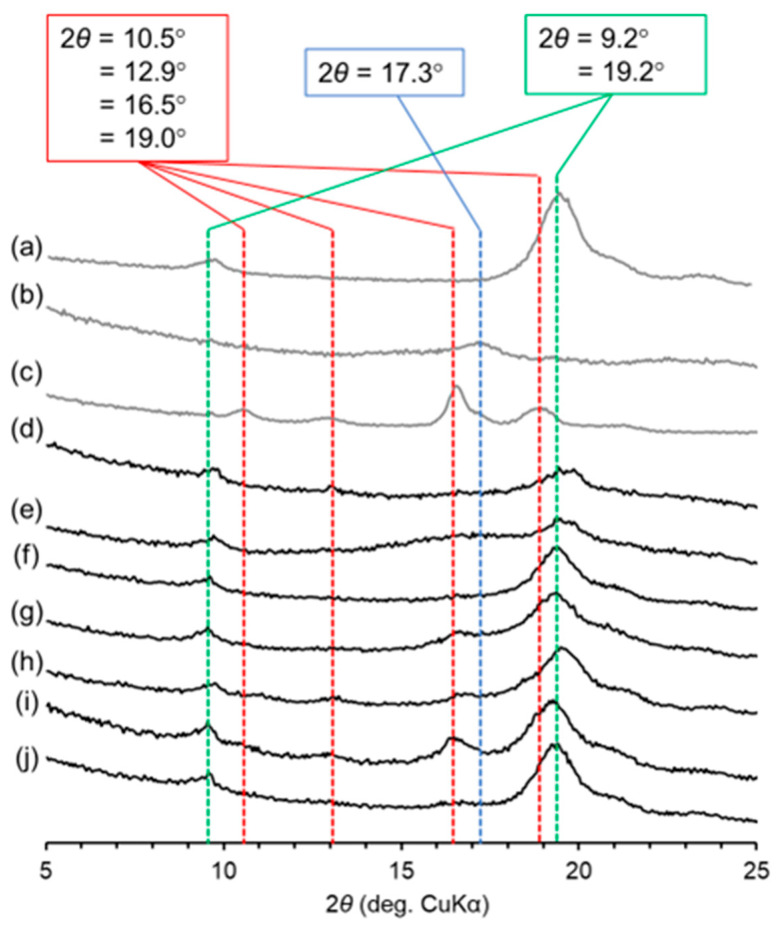
XRD profiles of (**a**) SD-ChNF (**b**) amylose, (**c**) 2-deoxyamylose, (**d**) amylose-grafted SD-ChNFs (entry 1), and P2D-amylose-grafted SD-ChNFs: (**e**) entry 2, (**f**) entry 3, (**g**) entry 4, (**h**) entry 5, (**i**) entry 6, and (**j**) 2-deoxyamylose-grafted SD-ChNFs for entry 7.

To explore the effect of P2D-amylose graft chains on crystalline structures of the obtained SD-ChNF derivatives, powder XRD analysis was performed. Figure 3 shows the XRD profiles of (a) SD-ChNF, (b) amylose, (c) 2-deoxyamylose, (d) amylose-grafted SD-ChNFs (entry 1), and P2D-amylose-grafted SD-ChNFs; (e) entry 2, (f) entry 3, (g) entry 4, (h) entry 5, (i) entry 6, and (j) 2-deoxyamylose-grafted SD-ChNFs (entry 7); pure amylose and 2-deoxyamylose samples were synthesized by the GP-catalyzed enzymatic homopolymerizations of Glc-1-P and d-glucan, respectively, from a maltotriose primer according to literature procedure [41,44]. In the XRD profiles of SD-ChNFs, the diffraction peaks ascribed to the crystalline structure of chitin were observed at 9.2 and 19.2° (Figure 3a) as detected in that of a raw chitin powder (Figure S2a). The XRD profiles of pure amylose and 2-deoxyamylose exhibited the characteristic patterns, respectively (at 2*θ* = 10.5/12.9/16.5/19.0° and 17.3° in Figure 3b,c) [40]. The XRD profile of the maltooligosaccharide-modified SD-ChNFs observed a similar pattern to that of a raw chitin powder and SD-ChNFs (Figure 3a and Figure S2), indicating that the modified maltooligosaccharide chains did not affect the crystalline structure of SD-ChNFs due to their short chain lengths. The main diffraction peaks at 9.2 and 19.2° in the XRD profile of the amylose-grafted SD-ChNFs (entry 1) were derived from the chitin crystalline structure, whereas other peaks were not obviously detected, since amylose exhibited low crystallinity (Figure 3d). The XRD profiles of P2D-amylose-grafted SD-ChNFs with Glc/2dGlc unit ratios = 26/74 and 50/50 (entries 2 and 3) also observed the patterns derived from the chitin crystalline structure, and did not mostly exhibit peaks derived from both amylose and 2-deoxyamylose (Figure 3e,f) because of the low crystallinity of the P2D-amylose graft chains with those unit ratios. These results are consistent with previous studies, which revealed the low crystallinity of the P2D-amyloses with similar unit ratios to those of the product of entry 2 (Glc/2dGlc unit ratio = 26/74), because of a random sequence of Glc/2dGlc units [40,41,43]. The XRD profiles of the products of entries 4–6 showed the characteristic peaks derived from 2-deoxyamyloses, prominently (Figure 3g–i). This is due to the formation of double-helices from the P2D-amylose graft chains as the 2dGlc unit ratios increase compared with the products of entries 2 and 3 [42]. On the other hand, the XRD profile of the 2-deoxyamylose-grafted SD-ChNFs (entry 7) largely exhibited the pattern derived from chitin crystalline structure, due to the low DP of the 2-deoxyamylose graft chains.

**Figure 4:**
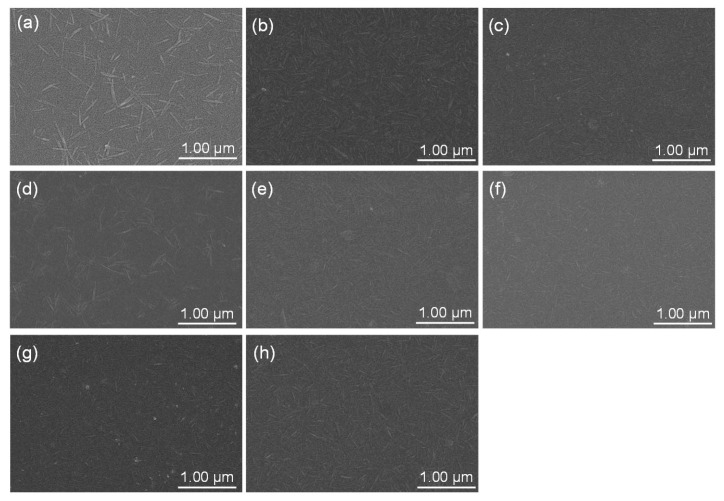
SEM images of spin-coated samples from (**a**) SD-ChNFs (**b**) amylose-grafted SD-ChNFs (entry 1), and P2D-amylose-grafted SD-ChNFs: (**c**) entry 2, (**d**) entry 3, (**e**) entry 4, (**f**) entry 5, (**g**) entry 6, and (**h**) 2-deoxyamylose-grafted SD-ChNFs (entry 7) dispersions.

**Figure 5:**
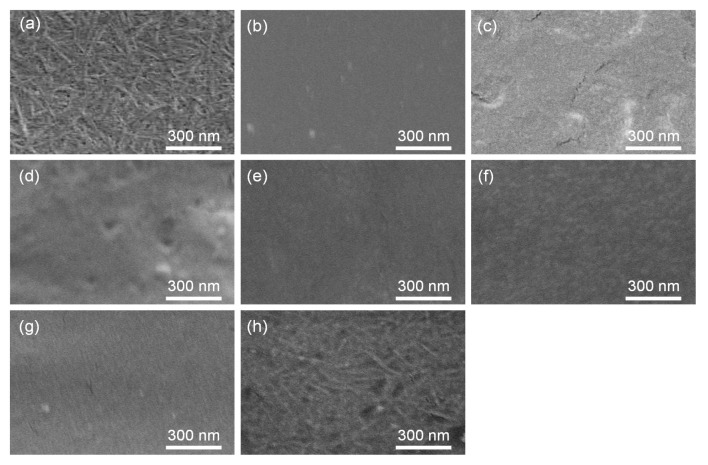
SEM images of cast films of (**a**) SD-ChNFs, (**b**) amylose-grafted SD-ChNFs (entry 1), and P2D-amylose-grafted SD-ChNFs; (**c**) entry 2, (**d**) entry 3, (**e**) entry 4, (**f**) entry 5, (**g**) entry 6, and (**h**) 2-deoxyamylose-grafted SD-ChNFs (entry 5).

To observe the morphological change of SD-ChNFs at the nanoscale level, by enzymatic grafting, DMSO dispersions of the products of entries 1–7, which were prepared by the ultrasonication of mixtures, were spin-coated on glass substrates and dried. Figure 4 depicts the scanning electron microscopic (SEM) images of the resulting samples from SD-ChNFs (a), amylose-grafted SD-ChNFs (b, entry 1), and P2D-amylose-grafted SD-ChNFs; (c) entry 2, (d) entry 3, (e) entry 4, (f) entry 5, (g) entry 6, and 2-deoxyamylose-grafted SD-ChNFs (h, entry 7). The image from SD-ChNFs observed the nanofiber structure (Figure 4a). In our previous study, the transmission electron microscopic image also supported the SD-ChNF morphology [22]. Similar morphologies were also detected in the other images (Figure 4b–h), indicating that the SD-ChNF structures were maintained by grafting the amylose derivatives on their surfaces. Figure 5 shows the surface morphologies of the films, which were prepared from mixtures of the products with DMSO via a simple casting method; (a) SD-ChNFs, (b) amylose-grafted SD-ChNFs (entry 1), and P2D-amylose-grafted SD-ChNFs; (c) entry 2, (d) entry 3, (e) entry 4, (f) entry 5, (g) entry 6, and (h) 2-deoxyamylose-grafted SD-ChNFs (entry 7). After the abovementioned DMSO dispersions, cast on silicone rubber cups, they were dried under ambient atmosphere at room temperature for 12 h to slowly evaporate the DMSO for the prevention of aggregation from SD-ChNF materials. The mixtures were subsequently dried under reduced pressure for 4 h at 60 °C for the complete removal of DMSO to form films with flat surfaces. The highly entangled nanofiber morphology was observed in the SEM image of the 2-deoxyamylose-grafted SD-ChNF film (Figure 5h), which was similar to that of SD-ChNFs (Figure 5a), owing to the low DP of the 2-deoxyamylose graft chains. On the contrary, the suppression of such nanofiber structures was seen, resulting in relatively smooth surfaces in the SEM images of the amylose- and P2D-amylose-grafted SD-ChNF films (Figure 5b–g). The root-mean-square surface roughness (*R*_RMS_) values were calculated from the laser microscopic images as these values represented the film surface roughness of the SD-ChNF, amylose-grafted SD-ChNF (entry 1), P2D-amylose-grafted SD-ChNFs (entries 2–6), and 2-deoxyamylose-grafted SD-ChNF (entry 7). Consequently, the *R*_RMS_ values were similar to an extent and showed no trend depending on the unit ratios and DPs of the graft chains on SD-ChNFs (*R*_RMS_ = 0.298–1.274 µm; see Figure S9). These results indicated that the amylose and P2D-amylose graft chains with high DPs had the possibility for covering the surfaces of SD-ChNFs.

**Figure 6:**
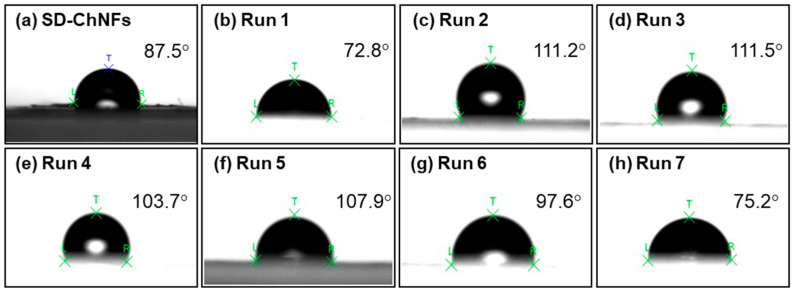
Water contact angles on film surfaces of (**a**) SD-ChNFs, (**b**) amylose-grafted SD-ChNFs (entry 1), and P2D-amylose-grafted SD-ChNFs; (**c**) entry 2, (**d**) entry 3, (**e**) entry 4, (**f**) entry 5, (**g**) entry 6, and (**h**) 2-deoxyamylose-grafted SD-ChNFs (entry 7). The water contact angles in images are the average values from four measurements.

To evaluate the hydrophobicity on the film surfaces of the P2D-amylose-grafted SD-ChNFs, the sessile drop contact angle with water as a probe has been measured, where the results are shown in Figure 6: (a) SD-ChNFs, (b) amylose-grafted SD-ChNFs (entry 1), and P2D-amylose grafted SD-ChNFs; (c) entry 2, (d) entry 3, (e) entry 4, (f) entry 5, (g) entry 6, and (h) 2-deoxyamylose-grafted SD-ChNFs (entry 7); the water contact angles are indicated as the average values by four measurements. The water contact angle values on the SD-ChNF and 2-deoxyamylose-grafted SD-ChNF films were not much different, that is, 87.5° and 75.2° (Figure 6a,h), indicating that the short 2-deoxyamylose chains on SD-ChNFs did not mostly affect their wettability. The grafting of the hydrophilic amylose chains resulted in the hydrophilicity of the amylose-grafted SD-ChNF film (water contact angle value = 72.8°) compared with the SD-ChNF film as shown in Figure 6b. The water contact angles on the P2D-amylose-grafted SD-ChNF films tended to increase according to the Glc/2dGlc unit ratios (from 26% to 62% of 2dGlc units in P2D-amylose chains) (entries 2–6, Figure 6c–g). Consequently, the P2D-amylose-grafted SD-ChNF film with the 26~50% 2dGlc unit ratios (entries 2 and 3) showed the highest water contact angle values (111.2~111.5°), suggesting their highest hydrophobicity, owing to the sufficient graft chain lengths and relatively high Glc/2dGlc unit ratios. Moreover, the low crystallinity of the graft chains might contribute to the hydrophobization of the SD-ChNF films. These results imply that the thermostable GP-catalyzed enzymatic grafting of the P2D-amylose chains achieved the efficient hydrophobization of nanofiber substrates.

## 3. Materials and Methods

### 3.1. Materials

Chitin powder (isolated from crab shells) was purchased from FUJIFILM Wako Pure Chemical Corporation (Wako), Osaka, Japan. The weight-average molecular weight was calculated by viscometrical analysis based on the literature method to be 7 × 10^5^ [47]. NaOH, methanol, NaBH_2_CN, K_2_PO_4_, D_2_O, DMSO-*d*_6_, and cyanotrihydroborate were purchased from Wako, Osaka, Japan. Acetic acid was purchased from Nacalai Tesque, Inc., Tokyo, Japan. An ionic liquid, AMIMBr, was synthesized by the reaction of 1-methylimidazole (Merk, Darmstadt, Germany) with 3-bromo-1-propene (Wako, Osaka, Japan) based on the method adapted from the literature [48]. DCl was purchased from Merk. Maltoheptatose was synthesized according to the literature procedure [49]. d-Glucal was synthesized by deacetylation of a commercially available *tri*-*O*-acetyl-d-glucal (Wako, Osaka, Japan) in sodium methoxide/methanol solution [40,42]. Glc-1-P (disodium salt hydrate) was purchased from Merk. Thermostable GP (from *Aquifex aeolicus* VF5) was supplied kindly by Dr. Takeshi Takaha (Sanwa Starch, Co., Ltd., Nara, Japan). Dialysis membrane (Spectra/Por^®^ 3, Waltham, MA, USA, MWCO = 3500) was purchased from REPLOGEN Corporation, Waltham, MA, USA. SD-ChNFs were prepared based on the literature procedure as described in Supplementary Materials [22,28,50,51].

### 3.2. Preparation of Maltooligosaccharide-Modified SD-ChNFs (Scheme 1a)

The PDA-ChNF film (54.7 mg, 0.089 unit mmol) with 1 M aqueous acetic acid (10 mL) was treated by ultrasonication using a homogenizer (Advanced-Digital Sonifier 450; 20 kHz, 400 W, Branson Ultrasonics Corporation, Danbury, CT, USA) for 10 min at room temperature to prepare a SD-ChNF dispersion. After maltooligosaccharide (5.15 g, 4.46 mmol) and NaBH_2_CN (1.12 g, 17.8 mmol) were mixed with the obtained dispersion, the mixture was stirred for 48 h at 60 °C. The obtained reaction mixture was filtered, and the residue was washed with water to yield the crude product. The residual gel-like material was added to water (10 mL) and subjected to ultrasonication using a homogenizer (Branson Advanced-Digital Sonifier 450; 20 kHz, 400 W) for 10 min at room temperature to produce a dispersion, which was lyophilized to yield maltooligosaccharide-modified SD-ChNFs (58.9 mg). ^1^H NMR (400 MHz, DCl/D_2_O = 5/1 (v/v)): δ 1.90 (s, AcOH), 2.25–2.30 (m, -COCH_3_), 3.14–4.08 (m, GlcNAc-H2,3,4,5,6, GlcN-H2,3,4,5,6, Glc-H2,3,4,5,6), 4.38 (d, Glc-H1β), 4.53 (d, GlcNAc-H1β), 4.66 (β(1→4)-GlcNAc-H1), 4.73 (d, GlcN-H1β), 4.87 (d, β(1→4)-GlcN-H1), 5.07 (d, Glc-H1α), 5.12 (d, GlcNAc-H1α), and 5.30 (d, GlcN-H1α).

### 3.3. Synthesis of Amylose-, P2D-Amylose-, and 2-Deoxyamylose-Grafted SD-ChNFs (Scheme 1b)

A typical experimental procedure was as follows (entry 2): A mixture of Glc-1-P (disodium salt, 143 mg, 550 μmol), d-glucal (80.3 mg, 550 μmol), and maltooligosaccharide-modified SD-ChNFs (23.8 mg, nonreducing ends; 0.0109 mmol) with 0.2 M Tris-acetate buffer (pH 6.9, 1.0 mL) containing KH_2_PO_4_ (7.45 mg, 0.0548 mmol) was maintained in the presence of thermostable GP (442 µL, 24 U) for 48 h at 60 °C with stirring. After the reaction mixture was stored in refrigerator overnight at 4 °C, the precipitated product was isolated by centrifugation (3500 rpm, 10 min), washed with water, and lyophilized to obtain the P2D-amylose-grafted SD-ChNFs (56.3 mg) in 58.3% yield based on amounts of the total Glc and 2dGlc residues present in the reaction system. ^1^H NMR (400 MHz, D_2_O/DCl/DMSO-*d*_6_ = 2/2/3 (*v*/*v*/*v*)): δ 1.53 (m, 2dGlc-H2 β-ax), 1.65 (m, 2dGlc-H2α-ax), 2.10 (m, 2dGlc-H2β-eq), 2.25 (m, 2dGlc-Hα-eq), 3.22–4.20 (m, Glc-H2,3,4,5,6, 2dGlc-H3,4,5,6), 4.65 (m, Glc-H1β), 4.95 (m, 2dGlc-H1β), 5.22 (s, Glc-H1α), and 5.27–5.42 (m, α(1→4)-Glc-H, 2dGlc-H1β).

### 3.4. Dispersion of Amylose-, P2D-Amylose-, and 2-Deoxyamyloses-Grafted SD-ChNFs on Glass Substrates for SEM Observation

Each product (1.0 mg, entries 1–7 in Table 1) was mixed with DMSO (10 mL). After the mixture was subjected to ultrasonication using a homogenizer (Branson Advanced-Digital Sonifier 450; 20 kHz, 400 W), the resulting dispersion was spin-coated (3000 rpm, 10 s) on a glass substrate, and slowly dried under reduced pressure for 12 h at 60 °C. The obtained sample was then subjected to SEM measurement.

### 3.5. Preparation of Films from Amylose-, P2D-Amylose-, and 2-Deoxyamylose-Grafted SD-ChNFs

Each product (20 mg, entries 1–7 in Table 1) was mixed with DMSO (1.0 mL). After the mixture was subjected to ultrasonication using a homogenizer (Branson Advanced-Digital Sonifier 450; 20 kHz, 400 W), the resulting dispersion was casted on a silicone rubber cup, and then dried under ambient atmosphere at room temperature for 12 h and, subsequently, under reduced pressure for 4 h at 60 °C to form a film.

### 3.6. Measurements

^1^H NMR spectra were recorded using an ECX400 instrument (JEOL, Akishima, Tokyo, Japan) for 64 scans at room temperature. SEM images were obtained using a Hitachi S-70 electron microscope (Hitachi High-Technologies Corporation, Tokyo, Japan) with an accelerating voltage of 15 kV. All samples were sputter-coated with platinum for 30 s with 30 mA using magnetron ion sputter coater (MSP-10, Vacuum Device Co., Ltd., Ibaraki, Japan) prior to SEM observation. Powder XRD measurements were carried out in the range of 5–40° using a PANalytical X’Pert Pro MPD (PANalytical B.V., EA Almelo, The Netherlands) with Nifiltered CuKα radiation (λ = 0.15418 nm). The crystalline index (CI) of the chitin crystal was estimated based on a method described in the literature using the following equation:

Cl (%) = [(*I*_110_ − *I*_am_)/*I*_110_] × 100 (1)

where *I*_110_ is the maximum intensity (arbitrary units) of the diffraction (110) at 2*θ* = 19.3° and Iam is the intensity of the amorphous diffraction in the same unit at 2*θ* = 16°. The film surface roughness was evaluated using laser microscopy (VK-X3000, Keyence, Osaka, Japan) (see Figure S9 and Supplementary Information). Water contact angles were measured with contact angle meter DropMaster 500 (Kyowa Interface Science Co., Ltd., Saitama, Japan), and we calculated the static contact angle using the *θ*/2 method incorporated in the FAMAS software version 1.9.2 (Kyowa Interface Science Co., Ltd., Saitama, Japan). The average values of water contact angles were determined by four measurements (mean ± standard deviation; see Table S1 in Supplementary Information).

## 4. Conclusions

We reported herein the successful hydrophobization on the SD-ChNF films by the thermostable GP-catalyzed enzymatic grafting of the P2D-amylose chains, as confirmed by the water contact angle measurements. The Glc/2dGlc unit ratios and chain lengths were crucial for achieving a quite high hydrophobicity. The ^1^H NMR analysis confirmed the structures of the desired SD-ChNF derivatives and their Glc/2dGlc unit ratios were dependent on the Glc-1-P/d-glucal feed ratios. The XRD analysis of the products revealed that the lengths and unit ratios of the grafted saccharide chains affected their entire crystalline structures. The water contact angle values of the films showed a tendency to increase above 100° depending on the Glc/2dGlc unit ratios. In conclusion, the P2D-amylose grafted SD-ChNFs functionally expresses strong hydrophobicity by controlling the Glc/2dGlc unit ratios in the graft chains on SD-ChNFs. Additional properties of the present SD-ChNF materials, such as the thermal property and biocompatibility, and the detailed hydrophobization mechanism by the incorporation of the P2D-amylose chains with optimal unit ratios, will be investigated in future study. The results in this study suggest that the present approach is a powerful procedure for the surface hydrophobization of chitin nanofibers and related substrates. Thus, this environmentally low-impact material design, which involves only the enzymatic grafting of biodegradable polysaccharides, could be applied to the hydrophobization of the other natural nanomaterials, leading to the unique functionalization of non-petroleum-derived products in the future.

## Figures and Tables

**Table 1:** Thermostable GP-catalyzed (co)polymerization of Glc-1-P/d-glucal from maltooligosaccharide-modified SD-ChNFs ^(a)^.

Entry	Feed Ratio (Glc_7_/Glc-1-P/d-glucal)	Unit Ratio (Glc/2dGlc) ^(c)^			DP ^(d)^	Content of Elongated Chain (wt%) ^(e)^	Thickness of Elongated Chain Layer/Radius of SD-ChNFs (=la/lb) ^(f)^
1	1/100/0	100	/	0	47	78.6	1.16
2 ^(b)^	1/50/50	74	/	26	25	67.2	0.75
3	1/25/75	50	/	50	30	68.0	0.78
4	1/20/80	48	/	52	28	67.0	0.74
5	1/15/85	43	/	57	27	63.3	0.65
6	1/10/90	38	/	62	22	61.4	0.61
7	1/0/100	0	/	100	3	15.0	0.08

^(a)^ Reaction was conducted at 60 °C for 48 h. ^(b)^ After the reaction, the solution was cooled at 4 °C for 24 h for precipitation. ^(c)^ Calculated from ^1^H NMR analysis. ^(d)^ Calculated from weight yield, Glc/2dGlc unit ratio, and degree of substitution (DS) of maltooligosaccharide primer on SD-ChNFs according to equations, reported in previous literature [33]. ^(e)^ Calculated from weight yield. ^(f)^ 
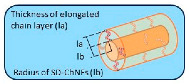
.

## Data Availability

The data are contained within the article and Supplementary Materials.

## Supplementary Materials

The following supporting information can be downloaded at: https://www.mdpi.com/article/10.3390/molecules30010016/s1, Preparation of SD-ChNFs, Figure S1: ^1^H NMR spectrum of the sample after dissolution of PDA-ChNF (degree of deacetylation = 31%) by acidic hydrolysis in D_2_O/DCl (5/1 vol/vol); Figure S2: XRD profiles of (a) chitin and (b) maltooligosaccharide-modified SD-ChNF; Figure S3: ^1^H NMR spectrum of the sample after selective hydrolysis and dissolution of amylose graft chains on SD-ChNFs (entry 1) in D_2_O/DCl/DMSO-*d*_6_ (3/3/2 vol/vol/vol); Figure S4: ^1^H NMR spectrum of the sample after selective hydrolysis and dissolution of P2D-amylose graft chains on SD-ChNFs (entry 3) in D_2_O/DCl/DMSO-*d*_6_ (3/3/2 vol/vol/vol); Figure S5: ^1^H NMR spectrum of the sample after selective hydrolysis and dissolution of P2D-amylose graft chains on SD-ChNFs (entry 4) in D_2_O/DCl/DMSO-*d*_6_ (3/3/2 vol/vol/vol); Figure S6: ^1^H NMR spectrum of the sample after selective hydrolysis and dissolution of P2D-amylose graft chains on SD-ChNFs (entry 5) in D_2_O/DCl/DMSO-*d*_6_ (3/3/2); Figure S7: ^1^H NMR spectrum of the sample after selective hydrolysis and dissolution of P2D-amylose graft chains on SD-ChNFs (entry 6) in D_2_O/DCl/DMSO-*d*_6_ (3/3/2 vol/vol/vol); Figure S8: ^1^H NMR spectrum of the sample after selective hydrolysis and dissolution of 2-deoxyamylose graft chains on SD-ChNFs (entry 7) in D_2_O/DCl/DMSO-*d*_6_ (3/3/2 vol/vol/vol); Figure S9: Laser microscopic images of cast films of (a) SD-ChNFs, (b) amylose-grafted SD-ChNFs (entry 1), and P2D-amylose-grafted SD-ChNFs; (c) entry 2, (d) entry 3, (e) entry 4, (f) entry 5, (g) entry 6, and (h) 2-deoxyamylose-grafted SD-ChNFs (entry 7); R_RMS_; the root-mean-square surface roughness; Table S1: Average and standard deviation values of water contact angles. References [22,28,50,51] are cited in the supplementary materials.
