# Supplementary material for: Hydrophobization of Chitin Nanofibers by Grafting of Partially 2-Deoxygenated Amyloses Through Enzymatic Approach
Source: Molecules. 2024 Dec 24;30(1):16. doi: 10.3390/molecules30010016 (PMC11722206; doi:10.3390/molecules30010016)
Supplement: Supplementary file 1 [file molecules-30-00016-s001.zip › molecules-3342773-supplementary.pdf]

## Supplementary Information

# Hydrophobization of Chitin Nanofibers by Grafting of Partially 2-Deoxygenated Amyloses Through Enzymatic Approach

Naoki Yamamoto, Masayasu Totani, Jun-ichi Kadokawa*

Graduate School of Science and Engineering, Kagoshima University, 1-21-40 Korimoto, Kagoshima 890-0065, Japan

**Preparation of SD-ChNFs** [1–4]

A mixture of chitin (0.120 g, 0.59 mmol) with AMIMBr (1.00 g, 4.92 mmol) was allowed to stand for 24 h at room temperature and then heated with stirring for 24 h at 100 °C to obtain a chitin ion gel (10 wt%). This gel was then immersed in methanol (30 mL) for 72 h at room temperature for regeneration, followed by ultrasonication (Branson 1510 (42 kHz, 70 W)) for 10 min to produce a self-assembled ChNF dispersion with methanol. The resulting dispersion was subjected to suction filtration to separate the ChNFs, which were washed with methanol and dried under reduced pressure to obtain a self-assembled ChNF film. After a mixture of the resulting ChNF film (0.120 g, 0.59 mmol) with aqueous NaOH (30 wt%, 20 mL) was heated for 24 h at 80 °C, the deacetylated material was isolated by suction filtration, immersed in water (30 mL) for 10 min via ultrasonication (Branson 1510 (42 kHz, 70 W)), filtered, washed with water, and dried under reduced pressure to obtain a PDA-ChNF film. $^1$H NMR (400 MHz, D2O+DCl (1/1 vol/vol), Fig. S1):

1.9 (AcOH), 2.1–2.3 (-$CH_3$), 2.2-2.5 (-NHAc), 3.0–4.0 (H2-6), 4.5–5.2 (H1). The degree of deacetylation (DDA) value of the product was calculated by the $^1$H NMR spectrum to be 31% for the total repeating units. A mixture of the PDA-ChNF film (80.0 mg, 0.41 mmol) with aqueous acetic acid (1 M, 20 mL) was ultrasonicated using a homogenizer (Branson Advanced-Digital Sonifier 450; 20 kHz, 400 W) for 10 min at room temperature to produce a SD-ChNF dispersion. For SEM observation, the sample, dispersed on a glass substrate, was prepared by spin-coating from the SD-ChNF dispersion, which was dried under reduced pressure for 24 h at 60 °C. For preparing a SD-ChNF film, the abovementioned dispersion was subjected to suction filtration and the residue was subsequently washed with water and dried under reduced pressure.

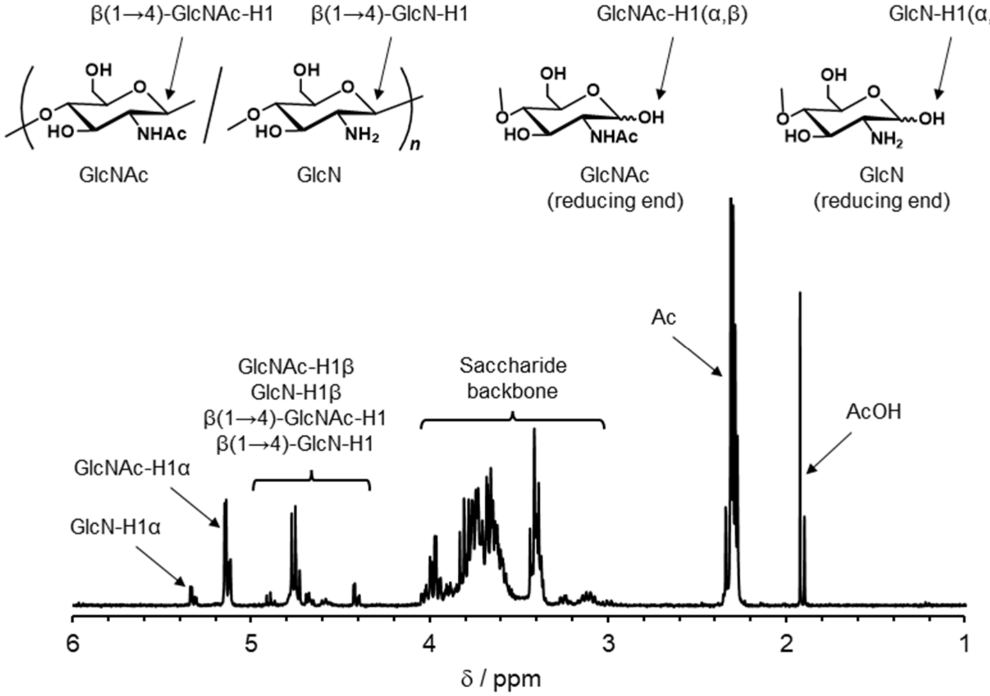


**Figure S1.** $^1$H NMR spectrum of the sample after dissolution of PDA-ChNF (degree of deacetylation = 31%) by acidic hydrolysis in $D_2O$/DCl (5/1 vol/vol).

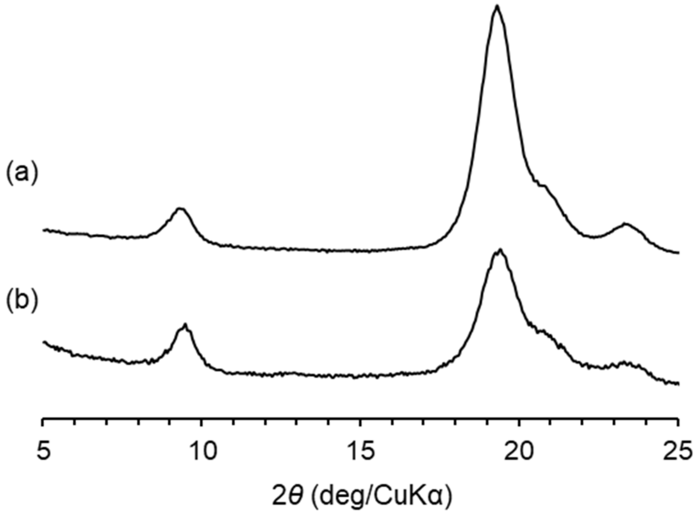


**Figure S2.** XRD profiles of (a) chitin and (b) maltooligosaccharide-modified SD-ChNF.

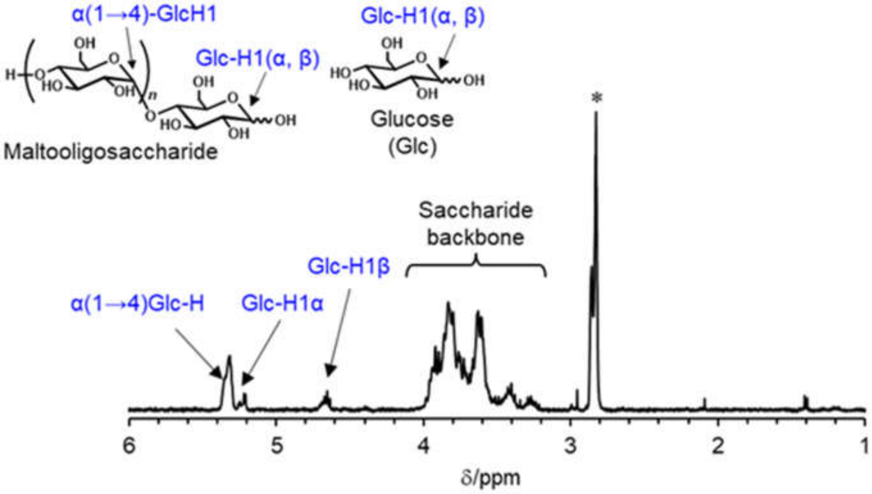


**Figure S3.** $^{1}$H NMR spectrum of the sample after selective hydrolysis and dissolution of amylose graft chains on SD-ChNFs (entry 1) in $D_2O$/DCl/DMSO-$d_6$ (3/3/2 vol/vol/vol).

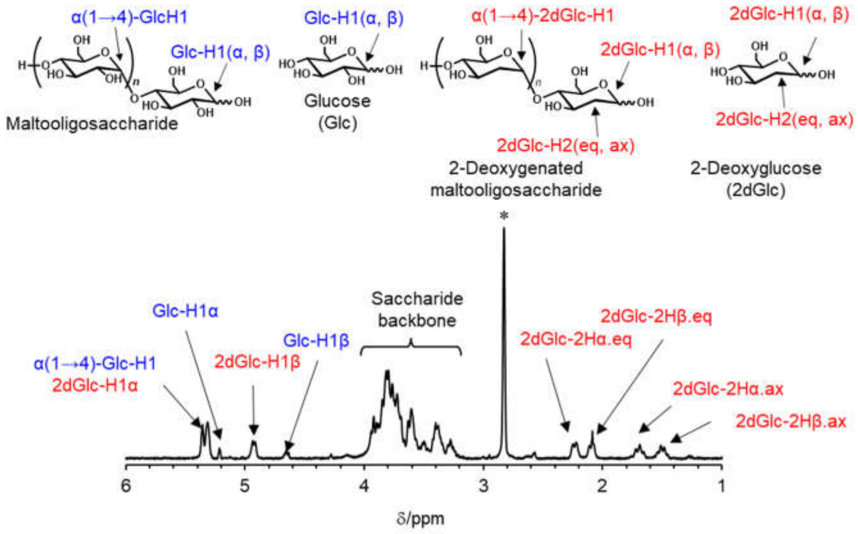


**Figure S4.** $^1$H NMR spectrum of the sample after selective hydrolysis and dissolution of P2D-amylose graft chains on SD-ChNFs (entry 3) in $D_2O$/DCl/DMSO-$d_6$ (3/3/2 vol/vol/vol).

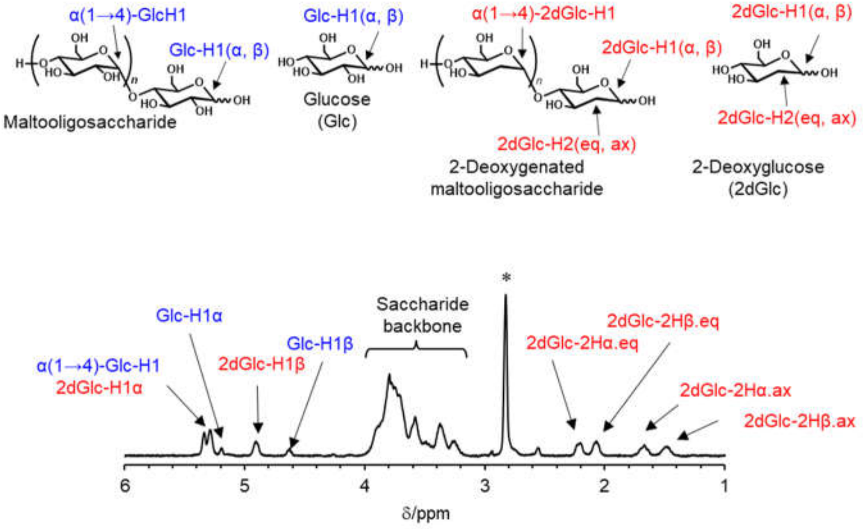


**Figure S5.** $^1$H NMR spectrum of the sample after selective hydrolysis and dissolution of P2D-amylose graft chains on SD-ChNFs (entry 4) in $D_2O$/DCl/DMSO-$d_6$ (3/3/2 vol/vol/vol).

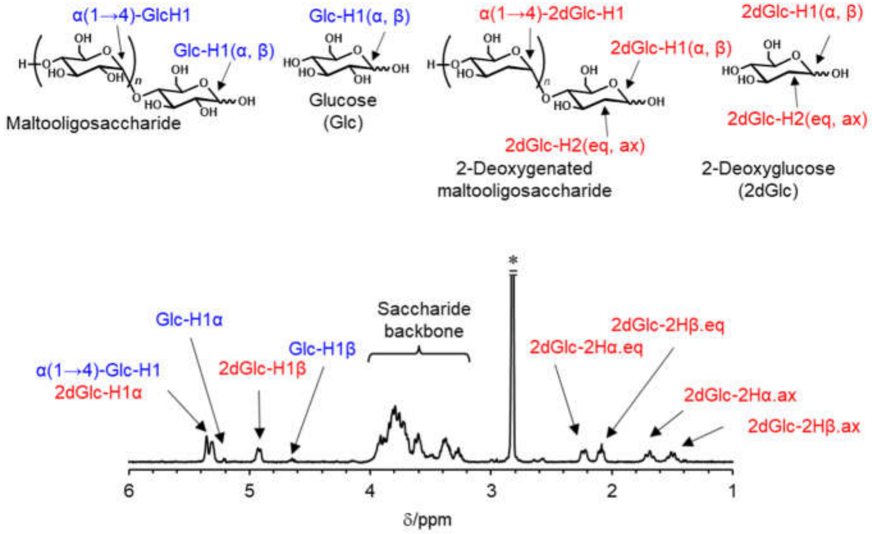


**Figure S6.** $^{1}$H NMR spectrum of the sample after selective hydrolysis and dissolution of P2D-amylose graft chains on SD-ChNFs (entry 5) in $D_2O$/DCl/DMSO-$d_6$ (3/3/2).

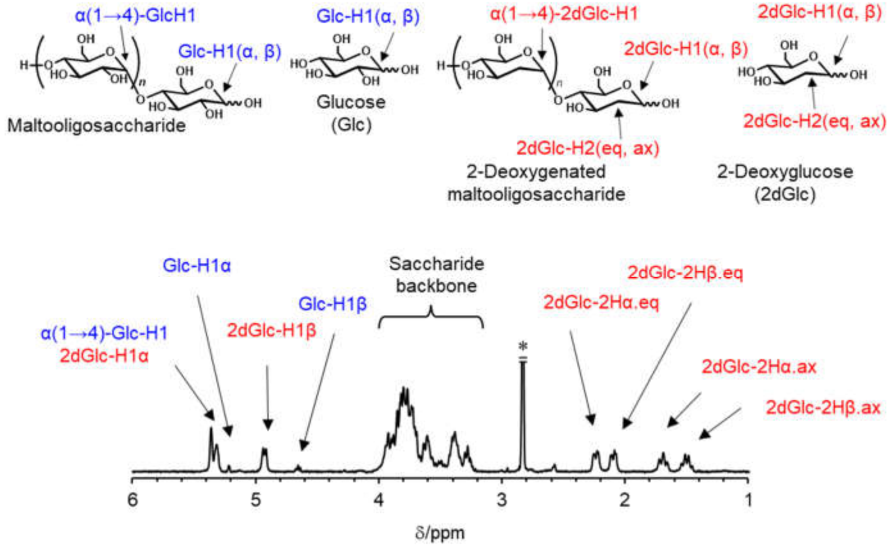


**Figure S7.** $^{1}$H NMR spectrum of the sample after selective hydrolysis and dissolution of P2D-amylose graft chains on SD-ChNFs (entry 6) in $D_2O$/DCl/DMSO-$d_6$ (3/3/2 vol/vol/vol).

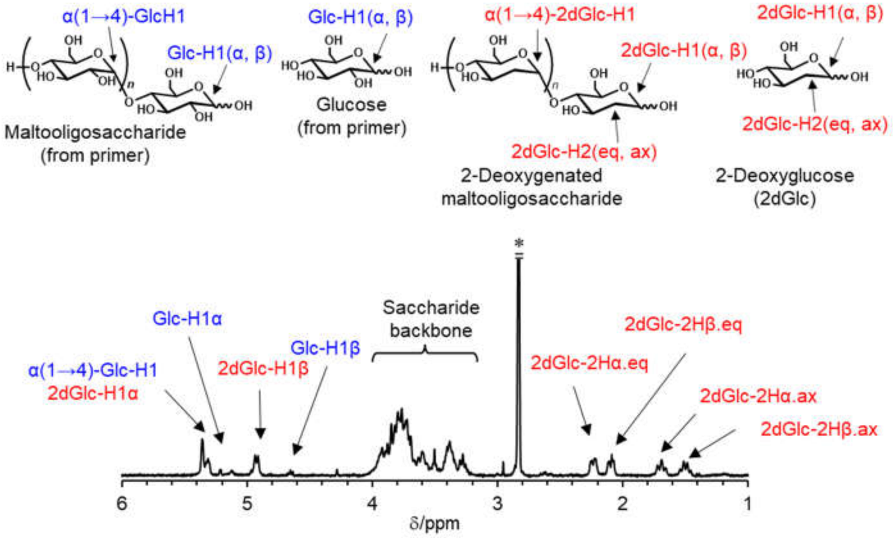


**Figure S8.** $^1$H NMR spectrum of the sample after selective hydrolysis and dissolution of 2-deoxyamylose graft chains on SD-ChNFs (entry 7) in $D_2O$/DCl/DMSO-$d_6$ (3/3/2 vol/vol/vol).

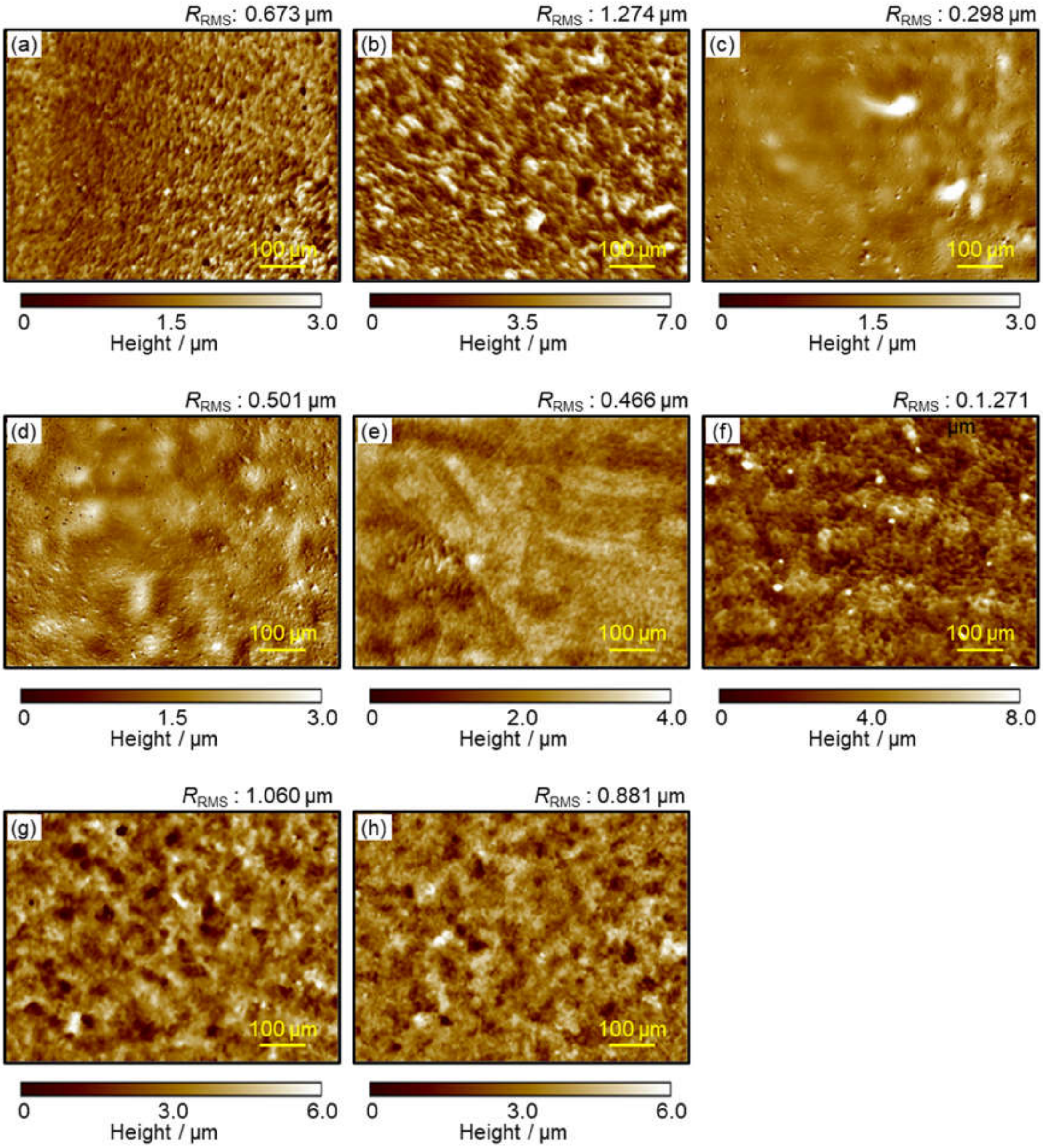


**Figure S9.** Laser microscopic images of cast films of (a) SD-ChNFs, (b) amylose-grafted SD-ChNFs (entry 1), P2D-amylose-grafted SD-ChNFs; (c) entry 2, (d) entry 3, (e) entry 4, (f) entry 5, (g) entry 6, and (h) 2-deoxyamylose-grafted SD-ChNFs (entry 7); $R_{RMS}$; the root-mean-square surface roughness

**Table.S1.** Average and standard deviation values of water contact angles[a)].

| Measurement times | SD-ChNFs | Entry 1 | Entry 2 | Entry 3 | Entry 4 | Entry 5 | Entry 6 | Entry 7 |
|---|---|---|---|---|---|---|---|---|
| 1 | 91.1 | 84.0 | 116.7 | 108.0 | 110.9 | 98.0 | 96.2 | 87.6 |
| 2 | 83.2 | 64.5 | 107.1 | 109.3 | 97.4 | 101.2 | 95.8 | 68.4 |
| 3 | 87.2 | 70.5 | 108.7 | 114.8 | 100.5 | 115.5 | 96.8 | 70.4 |
| 4 | 88.3 | 72.0 | 112.4 | 114.0 | 105.8 | 116.9 | 101.5 | 74.3 |
| Average | 87.5 | 72.8 | 111.2 | 111.5 | 103.7 | 107.9 | 97.6 | 75.2 |
| Standard deviation | 2.8 | 7.1 | 3.7 | 2.9 | 5.2 | 8.4 | 2.3 | 7.5 |

a) Entry 1; amylose-grafted SD-ChNF film, entries 2-6; P2D-amylose-grafted SD-ChNF films, entry 7; 2-deoxyamylose-grafted SD-ChNF film.

Reference:


1. J. Kadokawa, T. Setoguchi and K. Yamamoto, *Polym. Bull.*, 2013, **70**, 3279-3289.
2. J. Kadokawa, Y. Obama, J. Yoshida and K. Yamamoto, *Chem. Lett.*, 2018, **47**, 949-952.
3. J. Kadokawa, N. Egashira and K. Yamamoto, *Biomacromolecules*, 2018, **19**, 3013-3019.
4. T. Hashiguchi, K. Yamamoto and J. Kadokawa, *Carbohydr. Polym.*, 2021, **270**, 118369.
